# Low Circulation of Zika Virus, Cambodia, 2007–2016

**DOI:** 10.3201/eid2302.161432

**Published:** 2017-02

**Authors:** Veasna Duong, Sivuth Ong, Rithea Leang, Rekol Huy, Sowath Ly, Ugo Mounier, Teyputita Ou, Saraden In, Borin Peng, Sreymom Ken, Philippe Buchy, Arnaud Tarantola, Paul F. Horwood, Philippe Dussart

**Affiliations:** Institut Pasteur, Phnom Penh, Cambodia (V. Duong, S. Ong, S. Ly, U. Mounier, T. Ou, S. In, B. Peng, S. Ken, A. Tarantola, P.F. Horwood, P. Dussart);; National Center for Parasitology, Entomology, and Malaria Control, Phnom Penh (R. Leang, R. Huy);; GlaxoSmithKline Vaccines Research and Development, Singapore (P. Buchy)

**Keywords:** flavivirus, Zika virus, Flaviviridae, flavivirus, circulation, infection, diagnosis, viruses, Cambodia, vector-borne infections

## Abstract

We describe a retrospective study on circulation of Zika virus in Cambodia during 2007–2016 among patients with dengue-like symptoms and *Aedes aegypti* mosquitoes. Our findings suggest that Zika virus in Cambodia belongs to the Asia genotype, is endemic, has low prevalence, and has had low-level impact on public health.

Zika virus (family *Flaviviridae*, genus *Flavivirus*) is an arthropodborne virus mainly transmitted by the *Aedes* mosquito. Zika virus was first isolated in 1947 in Africa from rhesus macaques, and human illness was first recognized in Uganda in 1964 (*1*). This virus is known to cause various and nonspecific symptoms, such as fever, rash, arthralgia, headache, and conjunctivitis. In the second half of the 20th century, although the virus was detected in Africa and in Malaysia, only serologic evidence of Zika virus was reported in India, Thailand, Pakistan, Indonesia, Vietnam, and the Philippines (*2*). The first well-documented outbreak of Zika virus occurred in 2007 in Yap State, part of the Federated States of Micronesia (*3*), was followed by further outbreaks in the region (e.g., French Polynesia, New Caledonia, and Easter Island) in 2014 (*2*). Zika virus was detected in mainland South America in March 2015 in Brazil (*4*), subsequently spreading in South and Central America and most of the Caribbean over a short period (*2*). Before recent introduction and outbreaks of Zika virus in Singapore and Thailand (*5*), only sporadic autochthonous cases have been reported in Southeast Asia, whereas travel-associated cases from Zika-endemic countries have been detected in the United States, Europe, Southeast Asia, and Australia (*2*).

Zika virus infections have been associated recently with evidence of microcephaly related to transmission from mother to fetus during the first trimester of pregnancy (*6*). Moreover, other neurologic syndromes and nonvector modes of Zika virus transmission, including congenital, perinatal, and sexual, have been also described (*7*).

Cambodia’s National Dengue Surveillance System is a pediatric hospital–based syndromic surveillance system of suspected dengue cases managed by the Ministry of Health’s National Dengue Control Program (NDCP). Acute- and convalescent-phase samples from ≈5 patients randomly selected from each of the 5 sentinel sites in the NDCP system are collected and tested each week at Institut Pasteur du Cambodge (IPC) to ensure virologic diagnosis and surveillance (*8*). The first case of Zika virus infection in Cambodia was diagnosed in a 3-year-old child through community surveillance of acute fevers conducted during 2006–2010 in parallel to the NDCP sentinel system (*9*).

## The Study

We conducted a retrospective study by using samples in our biobank: 1) acute-phase serum and supernatant from NDCP collected during January 2007–July 2016; 2) acute- or convalescent-phase serum and supernatant samples collected by private clinics for dengue diagnosis during the same period; and 3) urine from dengue-negative patients and mosquito samples included in the DENFREE study conducted in Cambodia during 2012–2013 (Technical Appendix 1). The use of samples was approved by the National Ethics Committee for Health Research of Cambodia. We also tested 3,159 *Ae. aegypti* female mosquitoes negative for dengue infection by quantitative real-time reverse transcription PCR (qRT-PCR). In total, 2,400 serum samples and 173 supernatants from C6/36 cell cultures of serum from the dengue surveillance system, 270 urine samples from the DENFREE study, and 3,159 mosquitoes were tested for Zika virus by qRT-PCR (*10*). Positive results were confirmed by using conventional RT-PCR targeting the nonstructural protein 5 gene (*11*) and the Trioplex qRT-PCR kit (*12*). DNA products of conventional RT-PCR were sent for sequencing to a commercial sequencing facility (Macrogen, Inc., Seoul, South Korea). Serum samples were also tested for Zika virus IgM, dengue virus (DENV), and Japanese encephalitis virus (JEV) by using an in-house IgM capture ELISA (MAC-ELISA) (*13*). The interval between the date of symptom onset and the date of sampling ranged from 0 to 26 days (n = 1,922; mean 4.13 days, 95% CI 1.66–6.6 days).

**Figure 1:**
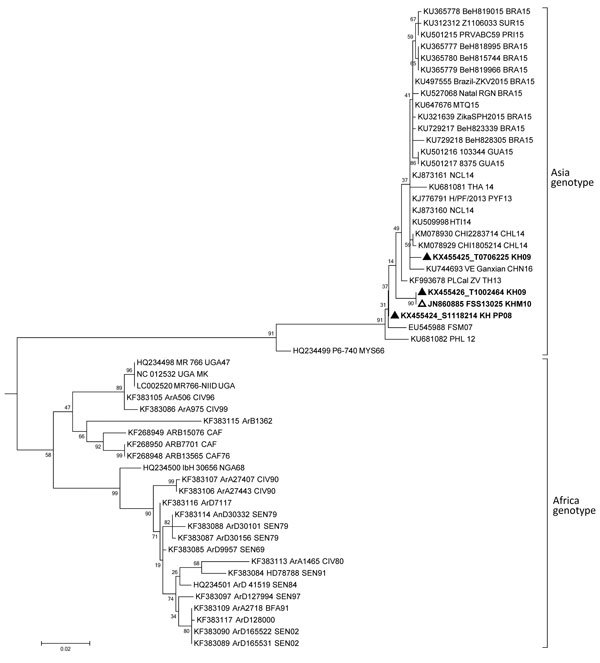
Phylogenetic tree of Zika virus partial nonstructural protein 5 gene of 3 strains detected from humans in Cambodia. The partial sequences of the nonstructural protein 5 gene (192–194 nt) generated from the PCR products obtained for each strain were analyzed and assembled by using CLC Main Workbench 5.5 package (CLC bio A/S, Aarhus, Denmark). MEGA6 (*14*) was used to perform multiple sequence alignment of Cambodia strains with Zika virus reference strains from Africa and Asia genotypes available in GenBank and phylogenetic analyses by using maximum-likelihood method using the general time reversible model with 1,000 bootstrap resampling. Spondweni virus (GenBank accession no. AF013406) was used to root the tree. Cambodia strains from this study are indicated by bold type and a black triangle, and the Cambodia strain isolated in 2010 is indicated by bold type and a white triangle. Scale bar indicates nucleotide substitutions per site.

Five human serum samples were positive for Zika virus by qRT-PCR; these samples were collected in 2007 (n = 1), 2008 (n = 1), 2009 (n = 2), and 2015 (n = 1) (Technical Appendix 2). No other samples, including urine and mosquito samples, were positive. Among the 5 positive samples, 3 were also positive by conventional RT-PCR. Phylogenetic analysis of partial sequences of the nonstructural protein 5 gene from human samples (1 from 2008 and 2 from 2009) showed that all 3 strains belonged to the Asia genotype. Zika virus strain S1118214 (GenBank accession no. KX455424) isolated in 2008 clustered closest with strains from the outbreak in Micronesia (2007); 1 of the strains isolated in 2009, T0706225 (accession no. KX455425), clustered closest with outbreak or endemic strains from New Caledonia (2014), Thailand (2014), China (2016), Chile (2014), and French Polynesia (2013); and the other strain isolated from 2009, T1002464 (accession no. KX455426), clustered closest with the Cambodia strain previously isolated in 2010 (*9*) (Figure 1). These findings suggests that Zika virus strains in Cambodia are closely related to strains causing outbreaks in the Pacific and South America and to endemic strains circulating in Asia; however, longer sequences are needed to confirm this finding.

The serologic study of 1,992 acute-phase serum samples showed that 16 samples from 2007 (n = 2), 2008 (n = 1), 2010 (n = 2), 2012 (n = 4), and 2015 (n = 7) were positive for Zika virus IgM, with no cross-reactivity with DENV and JEV, suggesting a presumptive diagnosis of recent infection. The interpretation of the 1,976 remaining samples is distributed as follows: 146 recent DENV infections; 4 recent JEV infections; 743 recent flavivirus infections (defined by IgM detection for >2 of 3 antigens tested [DENV, JEV, and Zika virus]); and 1,087 samples negative for antibodies for any of the 3 viruses tested.

**Figure 2:**
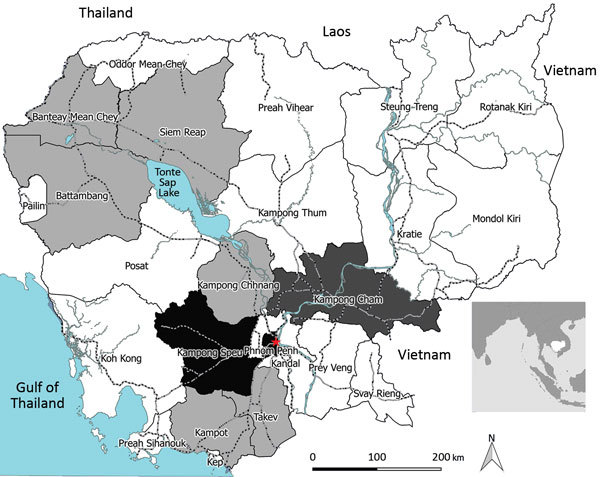
Geographic distribution of Zika virus in Cambodia. PCR- and IgM-positive cases were from 9 different provinces in north, central and south Cambodia. The 5 Zika virus–positive samples by quantitative real-time reverse transcription (qRT-PCR) in this study were distributed as follows: the 2007 (n = 1) sample was received from Kampong Cham province, and the other cases from 2008 (n = 1), 2009 (n = 2), and 2015 (n = 1) were from the Phnom Penh area (red star). The first case of Zika virus infection previously reported in Cambodia by conventional RT-PCR was diagnosed in a patient from Kampong Speu province (*9*). The 16 additional serum samples found to be positive for Zika virus IgM were, from oldest to the most recent, from Phnom Penh (2007, n = 1; 2008, n = 1; 2010, n = 2); Battambang (2007, n = 1; 2012, n = 1); Takeo (2012, n = 1); Kampong Speu (2012, n = 1); Kampot (2012, n = 1; 2015, n = 1); Kampong Chhnang (2015, n = 1); Banteay Meanchey (2015, n = 1); and Siem Reap (2015, n = 4) provinces. Light gray indicates provinces with IgM-positive cases, dark gray indicates province with PCR-positive cases, and black indicates provinces with PCR-positive and IgM-positive cases. Inset shows location of Cambodia in Southeast Asia.

The PCR- and IgM-positive Zika virus infections we detected came from 9 provinces in north, central, and south Cambodia (Figure 2). All 5 acute-phase samples that tested positive by RT-PCR were negative by MAC-ELISA. The convalescent-phase serum samples exhibited a pattern of recent flavivirus infection in 2 samples and recent Zika virus infection in 1 sample (Technical Appendix 2). The confirmed case in 2015 was considered to be an autochthonous case because no history of recent travel to Zika-endemic areas was reported by the patient. During January–May 2016, all suspected dengue samples that tested negative for DENV were also tested for Zika virus; no cases were detected, either by serology or RT-PCR.

We show that Zika virus circulated across Cambodia during the past 10 years with low intensity and limited effect on public health. No clear explanation exists for the low detection of Zika virus by RT-PCR or by MAC-ELISA, but it might be attributable to 1) the low incidence of Zika virus, which usually causes mild disease compared with DENV, in Cambodia; 2) possible bias in the selection of viremic cases, which were mainly reported in hospitalized suspected dengue patients and therefore were more severe; 3) less than ideal timing of specimen collection for the detection of Zika virus, which has been shown to be transient and frequently low-titer and too early for detection of Zika virus IgM on acute-phase serum samples; or 4) the difficulty in interpreting MAC-ELISA results owing to the cross-reactivity of IgM between DENV and JEV, which are widely present in Cambodia.

## Conclusions

The epidemiology of Zika virus is changing, and the risk for reintroduction in Cambodia is a real threat that should not be underestimated. Thailand, Vietnam, and Laos have reported cases since December 2015, and, local transmission has been observed recently in Singapore (*5*,*15*). *Ae. aegypti* and *Ae. albopictus* mosquitoes are widespread throughout Cambodia. The risk for reemergence or reintroduction of Zika virus should be monitored by extending the existing surveillance program to ambulatory suspected dengue patients and should be completed by a focal entomological study. Moreover, in the absence of a previously detected Zika outbreak in Cambodia, seroprevalence studies in the general population should help estimate the size of the population remaining susceptible to Zika infection.

Technical Appendix 1 Description of the DENFREE study conducted in Cambodia during 2012–2013.

Technical Appendix 2 Samples collected from 5 persons in Cambodia in 2007, 2008, 2009, and 2015 that tested positive for Zika virus by quantitative RT-PCR.
