# Supplementary material for: Low Circulation of Zika Virus, Cambodia, 2007–2016
Source: Emerg Infect Dis. 2017 Feb;23(2):296–9. doi: 10.3201/eid2302.161432 (PMC5324809; doi:10.3201/eid2302.161432)
Supplement: Technical Appendix 1 — Description of the DENFREE study conducted in Cambodia during 2012–2013. [file 16-1432-Techapp-s1.pdf]

Article DOI: http://dx.doi.org/10.3201/eid2302.161432

Low Circulation of Zika Virus, Cambodia, 2007–2016

Technical Appendix 1

The DENFREE Study

The aim of the DENFREE study was to document variation in DENV infectiousness of naturally infected humans across the spectrum of disease manifestations, including fully asymptomatic infections, and to verify the assumption that persons with asymptomatic infections are not infectious to mosquitoes (*1*). A secondary objective of this study was to assess the possibility of using saliva and urine samples instead of blood for dengue diagnosis (*2*).

Patients presenting with acute dengue-like illness between June and October of 2012 and 2013 were enrolled at Kampong Cham City Provincial Hospital and at two district hospitals of the Kampong Cham province. This province is the most populated with ≈1.7 million inhabitants and is ≈120 km northeast from Phnom Penh, the capital of Cambodia. Participant inclusion criteria were as follows: 1) age ≥6 months; 2) axillary temperature >38.0°C; 3) ≥2 of the following symptoms: headache, retro orbital pain, muscle pain, joint pain, rash, and any bleeding; and 4) written informed consent from the participant or a legal representative for participants <16 years old. DENV infection of hospitalized patients was confirmed by NS1 antigen detection using a commercial rapid diagnostic test followed by qRT-PCR on the plasma sample obtained during the acute febrile phase of disease (*3*). Patients with a confirmed DENV infection by NS1 antigen detection and/or qRT-PCR were considered dengue index cases (DICs) that initiated geographic cluster investigations. Serial plasma, urine and saliva samples were collected at several time-points between the day of admission to hospital until 3 months after the onset of fever in children with confirmed dengue disease.

Cluster participants were enrolled from family members at the DIC's household as well as from persons living in houses within a 200-m radius of the DIC's home or in the 20 closest houses when the population density of the area exceeded the logistical capacity of the field team.

Cluster participants were 2–40 years old and lived in villages located within 30 km from the provincial hospital. Cluster participants or representative for the participants <16 years old provided a written informed consent before enrollment. Exclusion criteria were as follows: 1) pregnancy or breastfeeding; 2) symptoms inconsistent with dengue and obvious nondengue acute infection (e.g., otitis media, pneumonia, and meningitis); or 3) known chronic illness. Blood samples were collected from household members and mosquitoes were collected from those households using motor aspirator for the detection of dengue virus.

References

1. Duong V, Lambrechts L, Paul RE, Ly S, Lay RS, Long KC, et al. Asymptomatic humans transmit dengue virus to mosquitoes. Proc Natl Acad Sci U S A. 2015;112:14688–93. PubMed http://dx.doi.org/10.1073/pnas.1508114112

2. Andries AC, Duong V, Ly S, Cappelle J, Kim KS, Lorn Try P, et al. Value of routine dengue diagnostic tests in urine and saliva specimens. PLoS Negl Trop Dis. 2015;9:e0004100. PubMed http://dx.doi.org/10.1371/journal.pntd.0004100

3. Hue KDT, Tuan TV, Thi HTN, Bich CTN, Anh HHL, Wills BA, et al. Validation of an internally controlled one-step real-time multiplex RT-PCR assay for the detection and quantitation of dengue virus RNA in plasma. J Virol Methods. 2011;177:168–73. PubMed http://dx.doi.org/10.1016/j.jviromet.2011.08.002
